# Analysis of UNESCO ESD Priority Areas’ Implementation in Romanian HEIs

**DOI:** 10.3390/ijerph192013363

**Published:** 2022-10-16

**Authors:** Corina-Ionela Dumitrescu, Georgiana Moiceanu, Razvan-Mihai Dobrescu, Mirona Ana Maria Popescu

**Affiliations:** 1 Department of Economics, University POLITEHNICA of Bucharest, 060042 Bucharest, Romania; 2 Department of Entrepreneurship and Management, University POLITEHNICA of Bucharest, 060042 București, Romania

**Keywords:** higher education institutions (HEIs), education for sustainable development (ESD), UNESCO priority areas for ESD, sustainable development

## Abstract

Higher education institutions (HEIs) are adopting sustainable development (SD) in their strategies for the future. The roadmap by UNESCO is the path to follow to reach success. The approach is different for every HEI, thus the objective of this paper is to analyze the current state of education for sustainable development activities provided by HEIs through the eyes of the academic community (responders category: professors, researchers, associate professors). The method to conduct the study was an interview that had 40 enclosed questions and a free part at the end where the responders could bring additional information to the study if they considered it necessary. All the interviews were transcribed and given a code (e.g., RHEI1, RHEI 29) in order to perform the analysis using descriptive statistics with the help of the program MS Office EXCEL. The results showed some areas where the activities provided by HEIs need improvement and also revealed promising aspects through partnerships. Making the values of SD known to the academic and local community will help fulfill the true potential for change and future development. Moreover, the analysis showed the need to educate educators and improve their digital skills and teaching methods/techniques in order to achieve sustainable development. Another result revealed the need for improvements in HEI curricula that will contribute to gaining those skills/abilities that emerging jobs should have.

## 1. Introduction

The agenda adopted by the United Nations member states in 2015 contains 17 sustainable development goals (SDGs) that are people- and planet-centered to achieve prosperity and peace through partnerships for sustainable development. Starting from the principle of solidarity, the agenda for sustainable development leaves no one behind, with the support of all individuals, communities, and countries. Lately, however, global threats such as inequalities and discrimination, poverty, unemployment, scarcity of resources, environmental degradation, climate change, and global warming, etc. have intensified as never before, so humanity must find new solutions to tackle them for communities/societies not to be at risk [1].

Since then, the Sustainable Development Report has been published annually. This report is a ranking in which nations are positioned due to their progress in achieving all 17 SDGs. For example, Finland has an overall score of 86.51, being in the first position of this ranking (163 countries) in 2022. A simple analysis suggests that Finland has progressed 86.51% of the way towards sustainable development, a level obtained by very good indicators for SDG1 (No Poverty), SDG4 (Quality Education), SDG7 (Affordable and Clean Energy), SDG9 (Industry, Innovation, and Infrastructure), and SDG10 (Reduced Inequalities). Furthermore, high progress in SDG4 (Quality Education) is a key factor for the overall score of the countries in the first positions of this ranking. Romania is in the 30th place in this ranking, with a total score of 77.7. This score was obtained by very good sub-indicators of SDG1 (No Poverty) and being on an ascending track for SDG6 (Clean Water and Sanitation). Unfortunately, the achievement of SDG4 (Quality Education) for Romania is on a descending trend. In this field, Romania registers an 84.52% participation rate in pre-primary organized learning (percentage of children aged 4 to 6) (with a slightly increasing rate from 2018 to 2019), 87.3% net primary enrollment rate (quite constant from 2018 to 2019), 88.5% lower secondary completion rate (quite the same in 2019 compared with 2018), and a 99.4% literacy rate in 2018 (percentage of the population aged 15 to 24) [2].

We must state that education for sustainable development means more than SDG4 (Quality Education). Quality education is about having equitable access to education and an increase in the number of individuals who obtain appropriate skills for easier insertion into the labor market, while education for sustainable development (ESD) is a learning process meant to train individuals to find solutions to major problems humanity confronts, with a holistic approach focused on empowering learners to act responsibly in order to achieve, simultaneously, environmental, economic, and social goals for the present and future generations, taking into consideration cultural diversity [3,4].

HEIs have an important role in implementing ESD. The objective of this paper is to analyze the current state of education for sustainable development activities provided by the public HEIs in Romania through the eyes of the academic community (responders category: professors, researchers, associate professors). This paper aims at identifying Romanian universities’ strengths and the opportunities related to ESD, but also the possible constraints, as well. The authors identify, through qualitative research, the Romanian higher education practice regarding ESD and the weaknesses that impede ESD in HEIs. It is also a good starting point for future research to identify the best ways of improving the communication between academic staff and university management so their actions are well known by all actors involved in a university. Romania is a major emergent country and this research can be considered a reference point for ESD in HEIs in other transition economies similar to Romania. Thus, the research hypothesis is that the academic community is aware of SDG implementation in the university and the necessity of implementation.

## 2. Literature Review

### 2.1. Education for Sustainable Development in Time

Most times, formal education towards sustainable development starts with great initiatives in primary school focusing on ecological/environmental education. After a few years of primary school, it seems that education for sustainable development stops suddenly. Many studies state that there are neither strategies, policies, or specific legislation for education for sustainable development in the higher education system, nor standards for integrating sustainability both in university lectures and professors’ training in higher education [5,6,7,8,9,10,11,12,13,14,15,16,17,18]. Each university is free to define its curriculum according to its domain and the experience of its human resources. At the same time, higher education institutions are in a continuous process of adapting their curriculum and practices taking into consideration the sustainability goals for 2030 [18,19].

In this context, environmental education (EE) might be considered the beginning of ESD which is from quite far in the past (1977) at the United Nations Conference on Sustainable Development, Stockholm, and the UNESCO and UNEP Intergovernmental Conference on Environmental Education, Tiblisi. It was stated then that environmental problems should be solved by individuals and societies through education in this field (knowledge, skills, attitudes, experience, involvement, and social responsibility) [20]. Additionally, education for sustainable development (ESD) is more transversal, starting with promoting quality education, building awareness for individuals and communities, and reshaping educational programs to focus on practical training. Therefore, starting from EE and moving towards ESD, humanity confronts three types of challenges: (a) sustainability concerns: people have to tackle diverse problems that cover a wide range of life aspects and threaten sustainability (from scarcity of resources to gender equality, from biodiversity loss to desertification, from health to energy issues); (b) values: people have to have a dynamic approach to customs, beliefs, and mentalities and to find solutions to combine past, present, and future when it comes to ESD; (c) placing ESD above all other education priorities at the international level (or at least comprising all of them): Millennium Development Goals, Education for all, United Nations Literacy Decade [21,22]. ESD can be defined as a frame of mind that can nurture the positive behavior needed for achieving sustainable development [23].

In 2022, 5 years after the United Nations Agenda was adopted, it became very clear that we need new tools for achieving sustainable development. UNESCO came up with a roadmap that identifies five priority areas to facilitate a way of achieving all 17 SDGs through education for sustainable development (ESD): (1) policy; (2) learning environments; (3) building the capacities of educators; (4) youth; and (5) local level action [24]. These areas mean a promise of contemporary societies to implement education for sustainable development.

**Figure 1:**
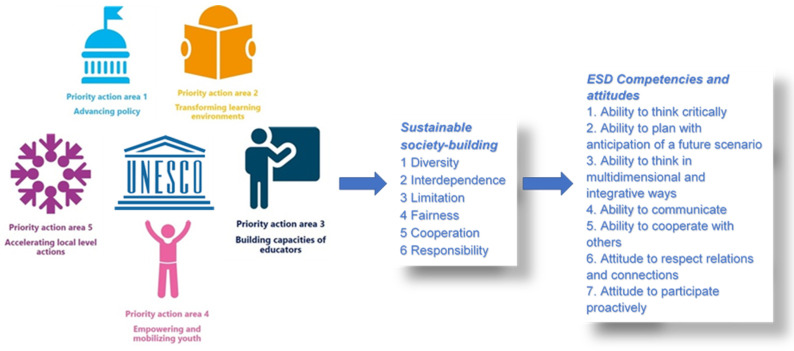
State of ESD in HEIs—general view. Source: Authors’ interpretation.

The UNESCO ESD roadmap is a vision of education for the present and future whose main objective is for individuals to obtain knowledge and competencies for a sustainable way of life: critical thinking, anticipatory thinking, multidisciplinary and integrative thinking, skills for effective communication, team working, the ability to build interpersonal relations, and emotional intelligence, as can be seen in Figure 1 [24,25].

### 2.2. Implementing ESD: The Great Outcome

Education is the trigger of all our actions. What we do depends on what we know and what we have learned through training, observation, and assimilation. Even if education is considered a necessity, after individuals obtain a study degree (usually a high school degree), many of them stop searching for a higher level of education [26]. Therefore, we consider that the implementation of ESD is vital for societies to be sustainable [24].

The implication of all stakeholders in all priority areas mentioned in the UNESCO ESD roadmap leads to a sustainable society based on diversity, freedom and interdependence, fairness, cooperation, and responsibility. From the perspective of sustainable development, all actors involved in education have to understand that the present and future are rather about skills/competencies than knowledge taught and learned. They have to know that solving complex problems, critical thinking, creativity, leadership, coordination, emotional intelligence, quick decision-making, communication, negotiation skills, and cognitive flexibility are very important in personal and professional lives and must consider them key factors for a sustainable life [24].

We consider that the main outcome of implementing ESD is to achieve a sustainable way of life. It is not simple to implement it because a global and transdisciplinary approach is needed. Moreover, it is not simple because in poor societies even education is considered difficult to implement, especially ESD. There are some points of view that state that ESD is what economics considers a normal or even luxury good [27]. Normal goods are those for which demand is higher and higher when the income of the consumer is higher and higher. In other words, the higher the economic/financial power of a society, the higher its capacity to implement ESD. We strongly believe that higher education institutions (HEIs) play a major role in this implementation even if they are different regarding the capacity for the implementation of ESD. Even countries are different when it comes to their performance in ESD [28].

The core of ESD is the concept of sustainable development, but it will be effective only if all stakeholders in education have a holistic approach and do not emphasize only one single side of sustainable development (economic, social, or environmental) depending on their background or mentalities [29,30,31].

The world needs a systemic framework for connecting sustainable development goals (SDGs) to educational outcomes so that societies may achieve a sustainable state. This systemic approach helps stakeholders (educators, students, policymakers in education, etc.) to identify problems and gaps in education, to develop curricula, and use pedagogical tools to have great social transformation from the perspective of sustainability. If Nelson Mandela said that “education is the most powerful weapon we can use to change the world”, we may admit that education for sustainable development is the most peaceful tool we have in order to obtain a sustainable life [32,33].

### 2.3. Higher Education Institutions (HEIs) and ESD

Higher education has an important role in achieving Quality Education (SDG4) and for other major goals, as well, from the perspective of 2030: No Poverty (SDG1), Good Health and Well-Being (SDG3), Gender Equality (SDG5), correlated with Reduced Inequalities (SDG10), Decent Work and Economic Development (SDG8), Sustainable Cities and Communities (SDG11), Responsible Consumption and Production (SDG12), Peace, Justice, and Strong Institutions (SDG16), and Partnerships for the Goals (SDG17). Therefore, HEIs have a complex mission nowadays: teaching and learning, researching (creating knowledge), and engaging in social progress [34].

To accomplish this triple role, HEIs have to adopt a sustainability-focused approach in their development strategies related to education, research, and social responsibility [35,36]. Consequently, HEIs will be able to be a great contributor to sustainable development considering a dynamic approach regarding education, research, and good governance, being an inspiration for all individuals and communities [37]. Such HEIs are integrative organizations that are able to select the best solutions and practices appropriate for sustainable development [38].

Sustainable development goals (SDGs) represent a suitable context for integrating education for sustainable development (ESD) into a higher education institution (HEI) [39,40]. In this framework, there are studies focused on general methods, strategies, and models for the implementation of ESD in HEIs aiming at incorporating sustainability into a general education curriculum [41,42]. Other studies put emphasis on triggers and barriers to the process [43,44,45] and ESD initiatives in formal education, university management, and extracurricular activities [46,47,48,49].

In this context, regarding ESD, only one course about sustainable development in the university curriculum is far from being sufficient to develop the competencies mentioned previously (solving complex problems, critical thinking, creativity, leadership, coordination, emotional intelligence, quick decision making, communication and negotiation skills, cognitive flexibility) [50]. A general and common framework is needed, in which curricula in HEIs are adapted and developed in such a manner to focus on ESD competencies integrated into teacher education programs, followed by a common framework for evaluating the efficiency of this process [51,52,53,54,55,56]. Furthermore, other studies evaluate the impact of HEIs on sustainable development focusing on the effects of HEIs outside its borders: economy at micro- and macroeconomic levels, policy-making, environment, culture, and demography, both in the short and long term. Short-term effects are considered direct (the knowledge the students acquire about sustainability) and the long-term effects are considered indirect (mentality changes and the adoption of a sustainable way of life) [57,58]. Furthermore, HEIs’ impact on sustainable development comes from either their organizational behavior (activities in education and research) or the individual behavior of their main stakeholders (students and academic staff) [57,58,59,60,61].

## 3. Materials and Methods

### 3.1. Research Methodology and Data Gathering

To provide the answers needed for the analysis of the current state of education for sustainable development activities provided by HEIs, in this paper the results of the research were made possible by conducting interviews addressed to the higher education institutions (HEIs) in Romania. The interview was addressed to all public HEIs in Romania. We contacted every higher education institution (HEI) in Romania by sending an email in which we explained the nature of the research and the request for their participation in the study. After this step, we received very few positive answers from the HEIs responsible for implementing sustainable development strategies or leaders that make decisions related to SD, but we received many positive answers from assistant professors, lecturers, and PhD students, and some from associate professors, professors, and researchers. After discussing all the positive answers and setting some limitations—mentioned in subchapter 3.3—we thankfully began the study with 46 people, one from every institution that accepted to be a part of our research study and fit the limitations. Thus, the number of respondents was 46 and could be included in these three categories: professors, associate professors (degrees for university teachers), and researchers (scientific researchers whose activity is based only on conducting research activities in various areas), as can be observed in Table 1.

Moreover, all responders have a minimum of 15 years of experience in the HEI they represent and were/are involved in the university academic community through a series of activities. The responders were contacted by the authors in order to set a date for the audio/video interview. After both parties agreed on a date, the interview took place as established. The interviews extended over a period of 3 months, from March to the end of May 2022. The responders, according to which HEI they represented, received a code: RHEI1, RHEI2, through to RHEI46. The codes were given to respect and offer confidentiality. Happily, no interview needed to be rescheduled, and all were conducted on time.

Therefore, the methodology used is qualitative, the main aim being to see if the five UNESCO priority areas were/are/will be considered by HEIs in Romania. This method was chosen due to the fact that examples can help bring more value to research and better express the responder’s additional information, thus the results and findings of this paper [62].

Because an opinion can offer great value and deeper meanings to this subject, education for sustainable development, the authors chose this approach and no other. The interview had close-ended questions, but at the end of the interview, any other information or comment that the responders considered useful could have been mentioned and their opinions are presented in the Results and Discussion section of the paper.

### 3.2. Interview Design

The interview design started from the five priority areas of ESD for 2030, identified by UNESCO, that can contribute to the achievement of 17 SDGs. Moreover, the interview design was conducted by analyzing the dimensions proposed by Lozano et al. [63] and the challenges that researchers already found [64,65] in connection to ESD adoption. Because we live in an era where time is of the essence for the interview design, the time to answer the questions was a factor considered in trying not to retain the responders for more than 20 min. The answers were collected using the existing platforms that allow for distance communication, such as Zoom, MS Teams, WebEx, Google Meets, and Skype. The number of responders for every platform is highlighted in Table 2.

A particular platform was used according to the desire of the responder. The interviews were conducted one on one, each question being addressed, and the answer was written down. The number of questions addressed was 40, and every question could have been answered with “yes/no/maybe/I don’t know”. The end of the interview was represented by comments/examples/opinions/information that the responder considered important for the scope of the research and in relation to any question answered before. At the end of all interviews, the results were collected, and analysis could be performed based on the answers. After each interview, the interviewer transcribed the answers in a document that was named according to the responders’ institution code (e.g., RHEI1). At the end of the interviews, after all the transcriptions were made, the data gathered and coded were analyzed using descriptive statistics with the help of the program MS Office EXCEL (Supplementary Material). So that the analysis can be enriched, as mentioned before, the examples given by the responders are also presented.

### 3.3. Study Limitations

Although the interviews were structured for people/employees from the HEIs responsible for implementing sustainable development strategies or leaders that make decisions related to SD, the responders that said yes to the interviews were researchers, professors, and associate professors. In this respect, participants such as assistants and lecturers were eliminated from being possible responders because it was important to have a person answering with a solid background and experience. Their background and experience were measured in the years spent in the HEI they represent and their activity within these years. Their involvement in the HEI’s activities such as projects, regulations, and academic issues such as admissions and final exams were criteria taken into consideration when considering conducting an interview, thus limiting us in our study implementation. In Romania, there are 53 public HEIs (including military universities) and 34 private HEIs; thus, considering this, another limitation was related to the type of HEI. The private universities were not questioned about ESD. This limitation was chosen because the focus and the development of both types of HEI are different. A study conducted first just on ESD in private HEIs and then a comparison between public and private HEIs could provide a perspective on the entire higher education system in relation to sustainable development.

## 4. Results

The search for answers regarding the analysis of the current state of education for sustainable development activities provided by the HEIs in Romania through the eyes of the academic community began by reaching out to the academic community, which proved to be challenging. The analysis began with agreeing to participate in the study and scheduling the interviews.

**Figure 2:**
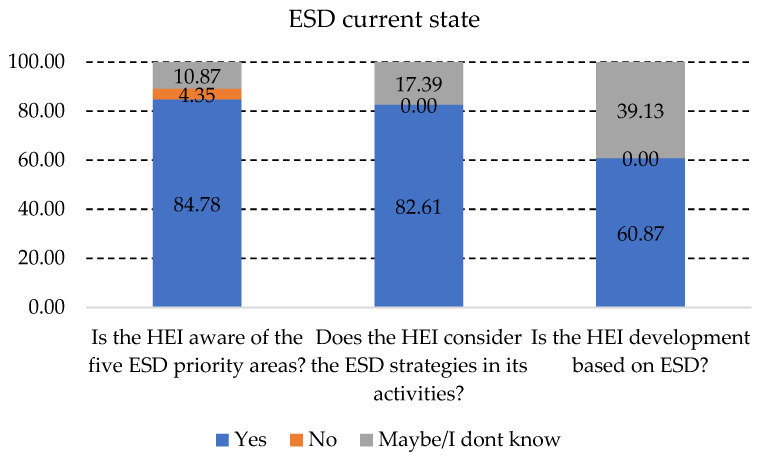
State of ESD in HEIs—general view.

The research done in the paper through the interviews started with a few questions related to the general aspects of ESD in HEIs. As can be seen in Figure 2, the five priority areas identified by UNESCO for ESD are known by more than 80% of the HEIs. Although the priorities are known, the development of the HEIs in relation to the priority key elements and whether they are applied or not are not publicly communicated, and the levels of implementation are not available to the public. The positive aspect of these questions is related to the fact that there were no “NO” answers for the second question, which can only represent that either the HEIs are using ESD strategies, or the responders are not aware whether the HEI they represent has an ESD strategy.

**Figure 3:**
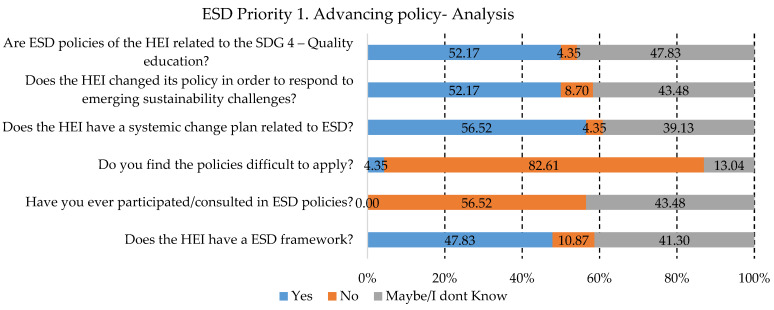
Priority area No. 1 results.

After this part was established, the questions related to the five priority areas for ESD, identified by UNESCO, were addressed to the responders. Thus, the first priority area, “Advancing policy” was analyzed, and the percentages of the answers are presented in Figure 3.

Sustainable development starts with policies that can provide the framework necessary for the future. The policymakers, along with practitioners, need to come together and find the best way to reshape the HEI system in order to answer the requirements for sustainable development. As can be seen from Figure 3, a percentage of 52.17% of HEIs started changing their policies. About 43.48% were not aware of this aspect in the HEI they represented. The most important aspect to mention for this priority is the fact that about 82.61% of the responders do not find the policies related to ESD difficult to apply, which leads us to the conclusion that most of them are willing to support educational change. One other encouraging aspect to mention is the quality of education that was taken into consideration for the ESD policies developed by the HEIs.

**Figure 4:**
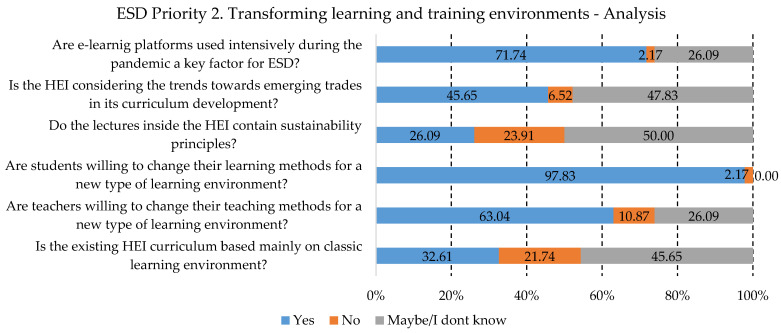
Priority area No. 2 results.

Continuing the analysis for the second priority area, “Transforming learning and training environments”, analyzed from the higher education point of view, the results can be seen in Figure 4. An important element that can lead to positive outcomes is the fact that approximately 98% of the students are willing to change their way of learning and, from the point of view of the responders, are open to new learning environments. Changing the way a class is taught or using technology becomes more appealing to students. Their involvement in class activities, individual research, and innovation contribute to the willingness of students to contribute to change. The change that has to be achieved, according to the results, is related to the curriculum, 33% of which is still using the classic learning environment.

**Figure 5:**
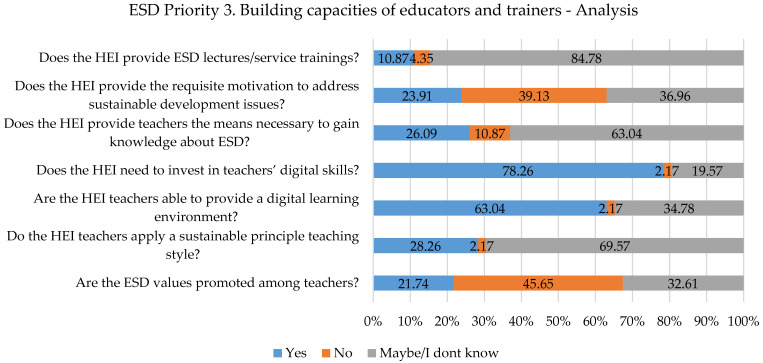
Priority area No. 3 results.

If we analyze Figure 5 to see the results for the third priority area, we can observe that there are many answers that do not provide a clear view of the aspects connected to it. The are many answers of “Maybe/I don’t know”, which can be a result of the fact that the responders are not part of the decisional authorities in the HEI or part of the structure responsible for sustainable development. One aspect that the responders mainly agreed on was the fact that teachers need digital skills and the HEIs should provide the means for it. Moreover, the change in the curriculum from the previous priority area is highlighted in this analysis because there are more “NO” answers than “YES” for applying sustainable principles to teaching styles. Along with special programs/lectures/training for teachers related to ESD, the values of ESD for SDGs should be promoted for a better commitment to achieving the goals for 2030.

When a current advancement is made at the expense of future generations, this is also known as unsustainable development. For instance, poor planning and resource exploitation can degrade the environment and harm ecosystems by producing waste and pollution. Mobilizing youth to contribute to changing an unsustainable pattern can help diminish the lack of appropriate education and awareness for sustainable development, with students being an important part of the higher education system.

**Figure 6:**
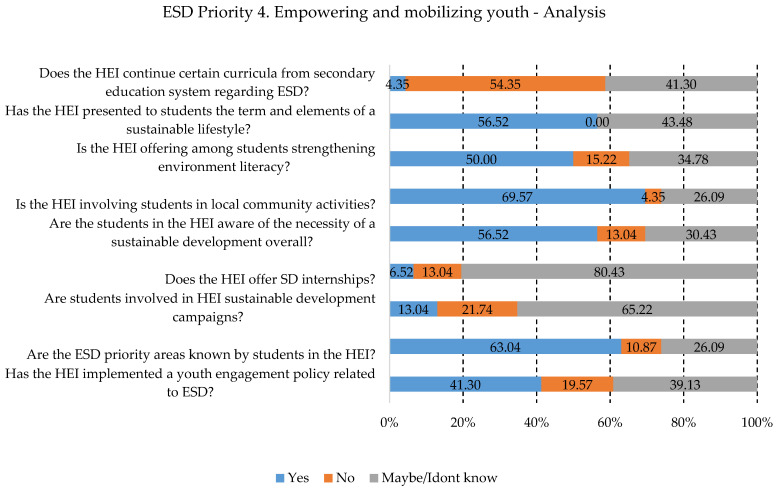
Priority area No. 4 results.

Also, a question that requires change inside the HEIs is related to SD internships. As can be seen in Figure 6, the responders are not aware of such activities. Moreover, the HEI does not continue the curriculum from secondary education, thus creating another gap that can only become bigger if the education system does not consider a continuous process through all stages of the education system.

**Figure 7:**
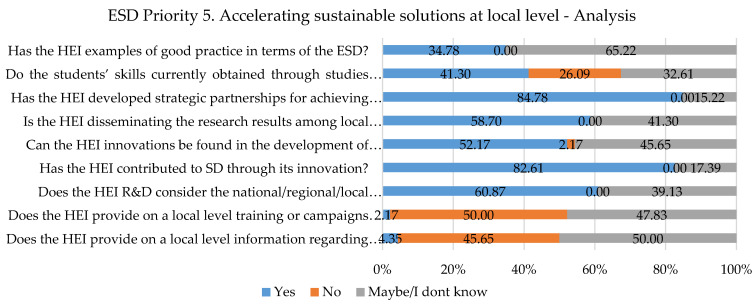
Priority area No. 5 results.

Continuing the analysis with the fifth and last priority area, it can also be observed that some questions do not reflect sustainable development. The work done on the curriculum and the teachers’ skills reflects the ability of the students to answer the challenges faced in contemporary society, whether we are looking at a local, regional, or national level. There are still 32.61% of the responders of the opinion that student skills are not what the business and social environments need. This environment requires students to have the ability to adapt to every situation, face the challenges that come, and be more proactive. All these can be achieved by engaging students in university activities and by putting them in situations that can need different characteristics in order to be solved. An encouraging aspect is that partnerships with public/private entities are being developed at a percentage of more than 84%. These results can be observed in Figure 7.

Moreover, this is not enough—the need for awareness and information campaigns are determined by activities that any HEI should consider at a local level in order to answer together for sustainable development goals. To ensure that the most recent theories and methods for sustainable development are applied to advance the local agenda, active cooperation between educational institutions and the community should be encouraged.

Demonstrating how people from all professions or stages of life can come together to study and take action in the search for sustainable futures is the idea of cooperation and education for all, the idea behind the “Accelerating sustainable solutions at local level” priority.

As mentioned before, the last part of the interview was addressed to information/opinions/aspects that the responders considered important to be mentioned. Most of the comments are on the direction of sustainable development and that it will require time to achieve the goals of the SDGs.

*RHEI5: “The HEI is acting in the direction of sustainable development. We are at the start of a department development that will be responsible for overseeing the implementation of policies, awareness campaigns, activities for teachers and students, that can all contribute to ESD.”*

*RHEI23: “Although we did not have a dedicated structure for sustainable development, we tackled these issues in every management plan developed for the university, and on top we changed and we are still in the process of changing some inside policies that can only lead to future development”*

*RHEI37: “We are taking some steps in the area of sustainable development, and we started this process through becoming a part of a consortium that can mean a good practice example, because through meeting/project, we can bring closer the element that can contribute to achieving the goals for every priority”.*

Other aspects that were mentioned by the responders were related to the willingness of teachers to change their teaching style. More than half of the respondents said that there are gaps between younger teachers compared to older ones. The desire to grow, to learn new ways of teaching, to apply various methods, or to change overall can be seen most of the time in teachers between 25 and 45 years old, unlike those over 45 years old who are used to doing things in a certain way.

*RHEI16: “A desire for a new teaching environment or to adopt another teaching method was expressed by our younger teaching staff. They mentioned the need for upgrading the environment that can also fulfill the needs of the socio-economic domain.”*

*RHEI41: “When you try to change the teaching environment of a professor, is like you are diminishing his entire life’s work. Most of the time, unfortunately, they do not see it as an evolution, but rather as a step back, and not just this, but as way of saying we don’t need you anymore.”*

It seems that from the free answers given, an opinion that is mainly the same for every responder is that teachers need to improve their digital skills. The COVID-19 pandemic contributed to this, forcing them to learn, but there is still a lack of experience related to digital skills.

*RHEI8: “Although most of us use electronic devices every day, this does not mean that everybody knows how to use them. In our HEI, a need for digital skills development which is on our short-term goals has been identified.”*

*RHEI19: “I am sorry to say this, but during the COVID-19 pandemic most of the teachers started using for the first time an e-learning platform, which was highly reflected in the quality of the teaching process. There were cases where everything would work just as it should have, but there were cases where it required training and even then, there still were elements unknown.”*

The last thing that needs to be mentioned is that some of the responders said that they are aware of the commitment that is needed for sustainable development, but the lack of action of the leading authorities enforces skepticism when considering the future of education for sustainable development.

*RHEI26: “I don’t think that the HEI alone will succeed in reaching the goals of the five priority areas, this can be possible just together with other leading authorities”*

*RHEI33: “The Education Ministry should clearly contribute to the objectives set for education regarding sustainable development. Not just that, but emerging trades should be taken into consideration when trying to improve the curriculum. Also, the business environment should be a part of this transformation, together with specialists from the socio-economic environment.”*

## 5. Discussion

Most European countries are on the right track when considering the transition to ESD, but there are still numerous obstacles to overcome, which can only mean that there should be more coordination across all education stakeholders to improve the transition to ESD, especially for low-performance countries [66].

The results reveal the need for greater attention on all aspects related to sustainable development. Even though significant steps to improve the education system towards sustainable development have been achieved, there is still room for improvement. As mentioned by the responders, not every institutional framework considers all five priority areas of ESD identified by UNESCO. Thus, as RHEI9 stated, “*the framework needs additional improvement to answer all areas and to be able to obtain all the aims desired also in the 17 SDGs*”. As a result, a framework with relevant measures should be taken into consideration for tracking the EU27’s progress towards efficient ESD. The elements that together constitute a priority chosen for each line of intervention are intended to capture both quantity and quality, and by including both, our notion differs from existing conceptual frameworks [28].

In Romania, access to education is unlimited because a sustainable society is built on a sustainable education system. Adults and children alike are motivated to advance their knowledge and abilities [67]. Thus, both priorities two and three can be fulfilled due to the education culture that is prepared for a long-lasting future, as the responders mentioned. RHEI31 said that the desire to change the future through building capacity and attractive learning environments is a part of the teachers’ values. Unfortunately, the budget allocations make it impossible to compare Romania to other European countries in this regard [68]. The need to empower the young generation and make them the factors of change can be achieved through the teacher’s capacity [62].

Although in previous research papers, the results revealed no interest in adding ESD competencies to the curriculum [69], now, through partnerships or by adding to their strategy in the University Charter for Sustainable Development, steps have been taken in this area [70]. The overcrowding of curricula, the apparent lack of significance to the curriculum, educational legitimacy, and limitations are cited as the key causes of institutions’ unwillingness to participate in sustainable education [62]. According to other researchers, for universities to become the drivers of sustainability issues and change agents, they must make sure that the needs of both the present and the future generations are better understood and built upon. This will allow professionals and teachers with expertise in SD to educate students of “all ages” in ways that will support the shift to “sustainable societal patterns,” as stated in frameworks [52,53,54,63,71,72]. Moreover, these findings are in agreement with our research and with the statements made by responders, that teachers must build their capacity and acquire the training they need to be able to contribute to change (just 27% of lectures include sustainability principles; a wide range of responders—more than 84%—are not aware of if the HEI is providing lectures related to SD; more than 39% of HEIs do not provide the requisite motivation necessary for addressing sustainable development issues). Moreover, teacher skills and professional improvement were addressed in this paper [73], revealing an interest in the study by the participants to discuss practices, teaching methods, and SD principles that can be integrated into ESD, which is in accordance with our findings—more than 63% of the teachers embracing change.

Regarding priority 4, it must be said that many young people in rural regions believe that migrating to the city is a better alternative for a better life, since they see the alternative as the more difficult option [74]. The UN, through its studies and activities, revealed that volunteering can be considered a method to develop pertinent skills and capacities able to guarantee that the Sustainable Development Goals are implemented in an inclusive and localized manner. As a result, in some regions of the world, there have been requests for the inclusion of volunteerism in the academic curricula, to encourage civic engagement and student leadership from an early age [75]. Our findings also show that unfortunately, the students are not that involved in local community activities or campaigns that can lead to sustainable development (priority 5 results). Moreover, this is consistent with results from other countries, for example, Sweden, where, according to a study, the results showed the need for ecosocial youth empowerment, which urges individuals to connect to social work practice, in order to have a greater understanding of both youth empowerment through SD and ecosocial work [76]. Moreover, our findings are in accordance with other results related to the development of sustainability abilities as strategies for navigating the diversity of the present era, which need to be aided by university degrees, youth empowerment, and community activity [42].

As mentioned before, HEIs can be the drivers of sustainability, not just through their teachers and students, but also through research. HEIs can shape a new path for the future of the world by delivering innovation to society [77]. According to researchers, a solution to benefit both the community and the HEI is “Living Labs” [59,60,61,77,78,79], which can ensure the connection between the socio-economic environment and the university in order to identify research and innovation opportunities, problems that could be solved, the development of strategic projects, etc. The partnerships appeared in our results as being addressed by the HEIs, which showed that over 84% have developed specific partnerships. Our findings are in accordance with UNESCO’s desire for the transformation of education, because by bringing together a range of stakeholders from most areas, a solution for sustainable education can be achieved and implemented [1,2]. Regarding research and innovation, just 58% and 52%, respectively, of the responders mentioned that their results can be found in the development of local-level areas, although more than 82% of the responders mentioned that their HEI’s innovation can contribute to sustainable development [80]. Moreover, awareness campaigns for the local/regional/national community can be considered an element of social marketing techniques that can lead to changes in thinking and behavior towards a specific social problem. Thus, a need that needs to be addressed, which resulted from research, is the lack of local-level training or campaigns for SD awareness, which, in turn, affects long-term SD change.

## 6. Conclusions

The SDGs’ implementation, socioeconomic recovery and progress, efforts to empower and develop societies in need, and the sustainability of any possible advancements prompted by the SDGs will all be impacted by the neglect of higher education. Without education that utilizes correct methods, tools, and objectives and equally targets all society segments, sustainable socioeconomic empowerment is difficult to attain.

Therefore, the analysis based on the five priority areas for ESD, identified by UNESCO in HEIs, sought to highlight the steps made by HEIs in creating an environment that can answer the challenges and meet the targets for sustainable development.

The study revealed a few implications and also several aspects that need improvement. Regarding the acceptance of change, both teachers and students are willing to embrace new skills and teaching abilities that contribute to the creation of new learning environments.

Regarding the implementation framework and adopting policies, there is still room for advancement and creation, mainly by decision-makers, which will affect HEIs’ policies in the future, in order to respond to emerging sustainability challenges.

Regarding the transformation of learning environments, it can be said that improvements in HEI curricula will help in answering the characteristics/skills/abilities needed for emerging traits, which, in the context of Industrial Revolution 4.0, automation through digitization, are subjected to permanent technological transformations, requiring a complex set of skills. The ability to adapt will bring society closer to sustainable development. For this objective, HEI teachers need to build their capacity, because their digital skills need improvement, and the HEIs need to provide the required motivation to address sustainable development issues; in one word, to “invest” in the academic community.

For the last priority, it can be said that the study revealed very few implications from HEIs in local community development, although they do contribute to solving issues through innovation. A good start can be seen through the high percentage of developed partnerships, which will lead to sustainable development.

Future research is recommended, and the study needs to be redone by applying the interviews to people inside the HEIs who are a part of developing sustainable development strategies or are involved in the decision-making process related to SD. The proper knowledge about ESD and its implementation inside HEIs will provide valuable insights as to the future steps that HEIs should take and, through best practices if there are any, how to take them.

This paper presented the situation of ESD in the academic community, with no authority point of view (professors, researchers, assistant professors) and the limited knowledge that these people have in relation to HEIs’ strategies for ESD. This article simply aimed to emphasize the current state and thus open the door for contemplation and future investigation, which the authors plan on realizing if decision-makers agree to be interviewed. By adopting sustainability into campus events including education, science, and infrastructure, one can implement sustainable university principles.

Furthermore, future research should examine whether some of the actions intended to help with achieving ESD really had an impact, and also to question and find if the adaptation of the curriculum changed the concept of SD for both teachers and students. Moreover, society overall is another path to follow for future analysis.

## Figures and Tables

**Table 1:** Responders’ classification.

Responders’ Category	No. of Participants
Associate professors	21
Professors	16
Researchers	9

**Table 2:** Communication platform.

Communication Platform	No. of Participants for Each Platform
Zoom	12
MS Teams	11
WebEx	6
Google Meets	8
Skype	9

## Data Availability

Not applicable.

## Supplementary Materials

The following supporting information can be downloaded at: https://www.mdpi.com/article/10.3390/ijerph192013363/s1, Interview architecture.

## References

[1] United Nations (2015). Transforming Our World: The 2030 Agenda for Sustainable Development.

[2] United Nations (2022). Sustainable Development Report 2022. From Crisis to Sustainable Development: The SDGs Roadmap to 2030 and Beyond.

[3] Leicht A., Heiss J., Byun W.J. (2018). Issues and Trends in Education for Sustainable Development.

[4] Unpacking Sustainable Development Goal 4: Education 2030. Guide—UNESCO Digital Library. https://unesdoc.unesco.org/ark:/48223/pf0000246300.

[5] European Commission Directorate-General for Education, Youth, Sport and Culture, Education and Training Monitor 2019. https://ec.europa.eu/education/sites/education/files/document-library-docs/volume-1-2019-education-and-training-monitor.pdf.

[6] Format for Reporting on Implementation of the UNECE Strategy for Education for Sustainable Development Phase III: 2011–2015. https://www.unece.org/fileadmin/DAM/env/esd/10thMeetSC/Documents/Czech_Republic.pdf.

[7] Format for Reporting on Implementation of the UNECE Strategy for Education for Sustainable Development within the Framework of the United Nations Decade of Education for Sustainable Development (2005–2014). https://www.unece.org/fileadmin/DAM/env/esd/Implementation/NIRs2010/6%20Denmark.pdf.

[8] Format for reporting on the Implementation of the UNECE Strategy for Sustainable Development (2017–2019). https://www.unece.org/fileadmin/DAM/env/esd/Implementation/NIR_2018/Estonian_ESD_NIR_final_2019.pdf.

[9] Finnish National Board of Education (2014). National Core Curriculum for Basic Education.

[10] Finland’s Ten Year Strategy and Guidelines 2006–2014 for Education for Sustainable Development. https://docplayer.net/18312677-Finland-s-ministry-of-education-a-national-strategy-and-guidelines-2006-2014-for-education-for-sustainable-development.html.

[11] Finland Reporting on the Implementation of the UNECE Strategy for Education for Sustainable Development (2017–2019). https://www.unece.org/fileadmin/DAM/env/esd/Implementation/NIR_2018/Finland_NIR_2018.pdf.

[12] Collectif Pour l’Intégration de la Responsabilité Sociétale et du Développement Durable Dans l’Enseignement Supérieur (CIRES). https://www.cirses.fr/.

[13] Rapport 2010 Sur La Mise En Oeuvre de la Stratégie de la Cee Pour L’éducation en vue du Développement Durable. https://www.unece.org/fileadmin/DAM/env/esd/Implementation/NIRs2010/34%20France.pdf.

[14] UNECE–10 Years of UNECE Strategy for Education for Sustainable Development. https://unece.org/DAM/env/esd/ESD_Publications/10_years_UNECE_Strategy_for_ESD.pdf.

[15] Higgins P., Scott W., Dillon J., Peters C. Education for Sustainable Development (ESD) in the UK-Current Status, Best Practice and Opportunities for the Future. https://www.researchgate.net/publication/336086683.

[16] Olsson D., Gericke D., Rundgen Chang S.-N. (2016). The Effect of Implementation of Education for Sustainable Development in Swedish Compulsory Schools—Assessing Pupils’ Sustainability Consciousness. Environ. Educ. Res..

[17] Economic Commision, for Europe United Nations Decade of Education for Sustainable Development (2005–2014) Good Practices in the UNECE Region Education for Sustainable Development in Action Good Practices N°2—2007UNESCO Education Sector. https://unesdoc.unesco.org/in/documentViewer.xhtml?v=2.1.196&id=p::usmarcdef_0000153319&file=/in/rest/annotationSVC/DownloadWatermarkedAttachment/attach_import,.

[18] Franco I., Saito O., Vaughter P., Whereat J., Kanie N., Takemoto K. (2018). Higher Education for Sustainable Development: Actioning the Global Goals in Policy, Curriculum and Practice. Sustain. Sci..

[19] Meschede C. (2020). The Sustainable Development Goals in Scientific Literature: A Bibliometric Overview at the Meta-Level. Sustainability.

[20] UNESCO (1977). Intergovernmental Conference on Environmental Education.

[21] Ndiaye A., Khushik F., Diemer A., Pellaud F. (2019). Environmetal Education for Sustainable Development: Challenges and Issues. Int. J. Humanit. Soc. Sci..

[22] Education for Sustainable Development: A Critical Reflexive Discourse on a Transformative Learning Activity for Business Students. https://link.springer.com/article/10.1007/s10668-022-02335-1.

[23] Bonnett M. (2002). Education for Sustainability as a frame of mind. Environ. Educ. Res..

[24] UNESCO (2020). Education for Sustainable Development: A Roadmap.

[25] Imara K., Altinay F. (2021). Integrating Education for Sustainable Development Competencies in Teacher Education. Sustainability.

[26] Kromydas T. (2017). Rethinking higher education and its relationship with social inequalities: Past knowledge, present state and future potential. Palgrave Commun..

[27] Rashid L. (2019). Entrepreneurship Education and Sustainable Development Goals: A literature Review and a Closer Look at Fragile States and Technology-Enabled Approaches. Sustainability.

[28] Momete D.C., Momete M.M. (2021). Map and Track the Performance in Education for Sustainable Development across the European Union. Sustainability.

[29] Klemeš J.J., Villas Bôas de Almeida C.M., Wang Y. (2013). Advancing Higher Education for Sustainable Development: International Insights. J. Clean. Prod..

[30] Sinakou E., Boeve-de Pauw J., Goossens M., Van Petegem P. (2018). Academics in the field of Education for Sustainable Development: Their conceptions of Sustainable Development. J. Clean. Prod..

[31] Kopnina H. (2020). Education for Sustainable Development Goals (ESDC): What is Wrong with ESDGs, and What Can We Do Better. Educ. Sci..

[32] Kioupi V., Voulvoulis N. (2019). Education for Sustainable Development: A Systemic Framework for Connecting the SDGs to Educational Outcomes. Sustainability.

[33] Mandela N. (2003). Lighting Your Way to a Better Future. Speech Delivered by Mr N R Mandela at Launch of Mindset Network Planetarium, University of the Witwatersrand Johannesburg South Africa. https://db.nelsonmandela.org/speeches/pub_view.asp?pg=item&ItemID=NMS909.

[34] Chankseliani M., McCowan T. (2021). Higher education and the Sustainable Development Goals. High. Educ..

[35] Owens T.L. (2017). Higher education in the sustainable development goals framework. Eur. J. Educ..

[36] Bessant S.E.F., Robinson Z.P., Ormerod R.M. (2015). Neoliberalism, new public management and the sustainable development agenda of higher education: History, contradictions and synergies. Environ. Educ. Res..

[37] Williams O., Swierad E.M. (2019). A Multisensory Multilevel Health Education Model for Diverse Communities. Int. J. Environ. Res. Public Health.

[38] Giesenbauer B., Müller-Christ G. (2020). University 4.0: Promoting the Transformation of Higher Education Institutions toward gSustainable Development. Sustainability.

[39] Filho W.L. (2018). Implementing Sustainability in the Curriculum of Universities. Approaches, Methods and Projects.

[40] Willats J., Erlandsson L., Molthan-Hill P., Dharmasasmita A., Simmons E., Filho W.L. (2018). A University Wide Approach to Embedding the Sustainable Development Goals in the Curriculum—A Case Study from the Nottingham Trent University’s Green Academy. Implementing Sustainability in the Curriculum of Universities.

[41] Cebrián G. (2018). The I3E model for embedding education for sustainability within higher education institutions. Environ. Educ. Res..

[42] Hill L.M., Wang D. (2018). Integrating sustainability learning outcomes into a university curriculum: A case study of institutional dynamics. Int. J. Sustain. High. Educ..

[43] Haigh M. (2005). Greening the university curriculum: Appraising an international movement. J. Geogr. High. Educ..

[44] Nomura K., Abe O. (2010). Higher education for sustainable development in Japan: Policy and progress. Int. J. Sustain. High. Educ..

[45] Milutinović S., Nikolić V. (2014). Rethinking higher education for sustainable development in Serbia: An assessment of Copernicus charter principles in current higher education practices. J. Clean. Prod..

[46] Lu S., Zhang H.-S. (2014). A comparative study of education for sustainable development in one British university and one Chinese university. Int. J. Sustain. High. Educ..

[47] Washington-Ottombre C., Bigalke S. (2018). An aggregated and dynamic analysis of innovations in campus sustainability. Int. J. Sustain. High. Educ..

[48] Mendoza J.M.F., Gallego-Schmid A., Azapagic A. (2019). A methodological framework for the implementation of circular economy thinking in higher education institutions: Towards sustainable campus management. J. Clean. Prod..

[49] Sima M., Grigorescu I., Bălteanu D. (2019). An overview of campus greening initiatives at universities in Romania. Int. J. Sustain. High. Educ..

[50] Merma-Molina G., Gavilán-Martín D., Baena-Morales S., Urrea-Solano M. (2022). Critical Thinking and Effective Personality in the Framework of Education for Sustainable Development. Educ. Sci..

[51] Baena-Morales S., Ferriz-Valero A., Campillo-Sánchez J., González-Víllora S. (2021). Sustainability Awareness of In-Service Physical Education Teachers. Educ. Sci..

[52] Rieckmann M. (2012). Future-oriented Higher Education: Which Key Competences Should Be Fostered through University Teaching and Learning?. Futures.

[53] Lambrechts W., Mulà I., Ceulemans K., Molderez I., Gaeremynck V. (2013). The integration of Competences for Sustainable development in Higher Education: An Analysis of bachelor Programs in Management. J. Clean. Prod..

[54] Lozano R., Merril M., Sammalisto K., Ceulemans k., Lozano F. (2017). Connecting Competences and Pedagogical Approaches for Sustainable Development in Higher Education: A Literature Review and Framework Proposal. Sustainability.

[55] Wiek A., Withycombe L., Redman C.L. (2018). Hey Competencies in Sustainability. A Reference Framework for Academic Program Development. Sustain. Sci..

[56] Cebrián G., Junyent M., Mulà I. (2020). Competencies in Education for Sustainable Development: Emerging Teaching and Research Developments. Sustainability.

[57] Findler F., Schönherr N., Lozano R., Stacherl B. (2019). Assessing the Impacts of Higher Education Institutions on Sustainable Development—Analysis of Tools and Indicators. Sustainability.

[58] Findler F., Schönherr N., Lozano R., Reider D., Martinuzzi A. (2019). Conceptualizing Sustainable Development Impacts on Higher Education Institutions. Int. J. Sustain. High. Educ..

[59] Boca G.D., Saraçli S. (2019). Environmental education and student’s perception, for sustainability. Sustainability.

[60] Urquidi-Martín A.C., Tamarit-Aznar C., Sánchez-García J. (2019). Determinants of the Effectiveness of Using Renewable Resource Management-Based Simulations in the Development of Critical Thinking: An Application of the Experiential Learning Theory. Sustainability.

[61] Lazzarini B., Pérez-Foguet A., Boni A. (2018). Key characteristics of academics promoting Sustainable Human Development within engineering studies. J. Clean. Prod..

[62] Sinden C.K. (2021). Incorporating Sustainability into the Academic Institution. Reinvention Int. J. Undergrad. Res..

[63] Lozano R., Ceulemans K., Alonso-Almeida M., Huisingh D., Lozano F.J., Waas T., Lambrechts W., Lukman R., Hugé J. (2015). A review of commitment and implementation of sustainable development in higher education: Results from a worldwide survey. J. Clean. Prod..

[64] Farinha C., Caeiro S., Azeiteiro U. (2020). Universities speak up regarding the implementation of sustainable development challenges. Int. J. Sustain. High. Educ..

[65] Aleixo A.M., Leal S., Azeiteiro U.M. (2018). Conceptualizations of sustainability in Portuguese higher education: Roles, barriers and challenges toward sustainability. J. Clean. Prod..

[66] Schleicher A. (2019). PISA 2018—Insights and Interpretations.

[67] OECD (2018). PISA 2015—Results in Focus. https://www.oecd.org.

[68] Eurostat (2019). Government Expenditure on Education. https://ec.europa.eu/eurostat/data/database.

[69] Dumitru D.E. (2017). Reorienting higher education pedagogical and professional development curricula toward sustainability—A Romanian perspective. Int. J. Sustain. High..

[70] Lazarov A.S., Semenescu A. (2022). Education for Sustainable Development (ESD) in Romanian Higher Education Institutions (HEIs) within the SDGs Framework. Int. J. Environ. Res. Public Health.

[71] Boström M., Andersson E., Berg M., Gustafsson K., Gustavsson E., Hysing E., Lidskog R., Löfmarck E., Ojala M., Olsson J. (2018). Condi-tions for transformative learning for sustainable development: A theoretical review and approach. Sustainability.

[72] Wals A.E. (2014). Sustainability in higher education in the context of the UN DESD: A review of learning and institutionalization pro-cesses. J. Clean. Prod..

[73] Biasutti M., Makrakis V., Concina E., Frate S. (2018). Educating academic staff to reorient curricula in ESD. Int. J. Sustain. High. Educ..

[74] Ma L., Chen M., Che X., Fang F. (2019). Farmers’ Rural-To-Urban Migration, Influencing Factors and Development Framework: A Case Study of Sihe Village of Gansu, China. Int. J. Environ. Res. Public Health.

[75] (2021). Sarjo Sarr, Empowering Youth for an Inclusive and Sustainable Development. https://www.unv.org/Success-stories/empowering-youth-inclusive-and-sustainable-development.

[76] Chang E., Sjöberg S., Turunen P., Rambaree K. (2022). Youth Empowerment for Sustainable Development: Exploring Ecosocial Work Discourses. Sustainability.

[77] Purcell W.M., Henriksen H., Spengler J.D. (2019). Universities as the engine of transformational sustainability toward delivering the sustainable development goals “Living labs” for sustainability. Int. J. Sustain. High. Educ..

[78] Waheed M.H. (2017). A Revolution for Post-16 Education—Part 2: How Do Living Labs Work?. www.sustainabilityexchange.ac.uk/files/living_labs_project_part_2.pdf.

[79] Purcell W.M., Sharp L., Chahine T. New Governance Models for Entrepreneurial Universities: A Conceptual Framework. Proceedings of the 2017 University-Industry Engagement Conference: From Best Practice to Next Practice—Asia-Pacific Opportunities and Perspectives.

[80] Stephen J.C., Hernandez M.E., Roman M., Graham A.C., Scholz R.W. (2008). Higher education as a change agent for sustainability in different cultures and contexts. Int. J. Sustain. High. Educ..

