# Supplementary material for: Analysis of UNESCO ESD Priority Areas’ Implementation in Romanian HEIs
Source: Int J Environ Res Public Health. 2022 Oct 16;19(20):13363. doi: 10.3390/ijerph192013363 (PMC9602468; doi:10.3390/ijerph192013363)
Supplement: Supplementary file 1 [file ijerph-19-13363-s001.zip › ijerph-1932099-supplementary.pdf]

International Journal of
Environmental Research and Public Health

MDPI

Article

Analysis of UNESCO ESD priority areas in Romanian HEIs

Corina-Ionela Dumitrescu [1], Georgiana Moiceanu [2]*, Razvan-Mihai Dobrescu [1] and Mirona Ana Maria Popescu[2]

Supplementary material

Interview architecture
Date of the interview: ……………………………………

Education for sustainable development (ESD) is UNESCO's education sector response to the urgent and dramatic challenges the planet faces. The collective activities of human beings have altered the earth's ecosystems so that our very survival seems in danger because of changes more difficult to reverse every day. Thus, the Priority action areas proposed are:

1. Priority action area 1: Advancing policy
2. Priority action area 2: Transforming learning environments
3. Priority action area 3: Building capacities of educators
4. Priority action area 4: Empowering and mobilizing youth
5. Priority action area 5: Accelerating local level actions

Taking into consideration the priority action areas of UNESCO on ESD, please answer the questions below as accurate as possible.
Thank you kindly.

General ESD aspects

1. Is the HEI aware of the five ESD priority areas?
   Yes
   No
   Maybe /I don't know
2. Does the HEI consider the ESD strategies in its activities?
   Yes
   No
   Maybe /I don't know
3. Is the HEI development based on ESD?
   Yes
   No
   Maybe /I don't know

Advancing policy

4. Does the HEI have a ESD framework?
   Yes

No
Maybe /I don't know

5. Have you ever participated/consulted in ESD policies?
Yes
No
Maybe /I don't know
6. Do you find the policies difficult to apply?
Yes
No
Maybe /I don't know
7. Does the HEI have a systemic change plan related to ESD?
Yes
No
Maybe /I don't know
8. Does the HEI changed its policy in order to respond to emerging sustainability challenges?
Yes
No
Maybe /I don't know
9. Are ESD policies of the HEI related to the SDG 4 – Quality education?
Yes
No
Maybe /I don't know

Transforming learning and training environments

10. Is the existing HEI curriculum based mainly on classic learning environment?
Yes
No
Maybe /I don't know
11. Are teachers willing to change their teaching methods for a new type of learning environment?
Yes
No
Maybe /I don't know
12. Are students willing to change their learning methods for a new type of learning environment?
Yes
No
Maybe /I don't know
13. Do the lectures inside the HEI contain sustainability principles?
Yes
No
Maybe /I don't know
14. Is the HEI considering the trends towards emerging trades in its curriculum development?

Yes

No

Maybe /I don't know

15. Are e-learning platforms used intensively during the pandemic a key factor for ESD?

    Yes

    No

    Maybe /I don't know

Building capacities of educators and trainers

16. Are the ESD values promoted among teachers?

    Yes

    No

    Maybe /I don't know
17. Do the HEI teachers apply a sustainable principle teaching style?

    Yes

    No

    Maybe /I don't know
18. Are the HEI teachers able to provide a digital learning environment?

    Yes

    No

    Maybe /I don't know

19. Does the HEI need to invest in teachers' digital skills?

    Yes

    No

    Maybe /I don't know
20. Does the HEI provide teachers the means necessary to gain knowledge about ESD?

    Yes

    No

    Maybe /I don't know
21. Does the HEI provide the requisite motivation to address sustainable development issues?

    Yes

    No

    Maybe /I don't know
22. Does the HEI provide ESD lectures/service trainings?

    Yes

    No

    Maybe /I don't know

Empowering and mobilizing youth

23. Has the HEI implemented a youth engagement policy related to ESD?

    Yes

    No

Maybe /I don't know

24. Are the ESD priority areas known by students in the HEI?
Yes
No
Maybe /I don't know
25. Are students involved in HEI sustainable development campaigns?
Yes
No
Maybe /I don't know
26. Does the HEI offer SD internships?
Yes
No
Maybe /I don't know
27. Are the students in the HEI aware of the necessity of a sustainable development overall?
Yes
No
Maybe /I don't know
28. Is the HEI involving students in local community activities?
Yes
No
Maybe /I don't know
29. Is the HEI offering among students strengthening environment literacy?
Yes
No
Maybe /I don't know

30. Has the HEI presented to students the term and elements of a sustainable lifestyle?
Yes
No
Maybe /I don't know
31. Does the HEI continue certain curricula from secondary education system regarding ESD?
Yes
No
Maybe /I don't know

Accelerating sustainable solutions at local level

32. Does the HEI provide on a local level information regarding ESD?
Yes
No
Maybe /I don't know
33. Does the HEI provide on a local level training or campaigns for SD awareness?
Yes

No

Maybe /I don't know

34. Does the HEI R&D consider the national/regional/local social/environmental problems?

Yes

No

Maybe /I don't know

35. Has the HEI contributed to SD through its innovation?

Yes

No

Maybe /I don't know

36. Can the HEI innovations be found in the development of local level areas?

Yes

No

Maybe /I don't know

37. Is the HEI disseminating the research results among local community actors involved in SD change?

Yes

No

Maybe /I don't know

38. Has the HEI developed strategic partnerships for achieving ESD?

Yes

No

Maybe /I don't know

39. Do the students' skills currently obtained through studies correspond to contemporary societal challenges?

Yes

No

Maybe /I don't know

40. Has the HEI examples of good practice in terms of the ESD?

Yes

No

Maybe /I don't know

Please feel free to add any other measures/opinions/facts/information concerning ESD aspects in your HEIs that you consider relevant.

………………………………………………………………………………………………..
………………………………………………………………………………………………..
………………………………………………………………………………………………..
………………………………………………………………………………………………..
………………………………………………………………………………………………..
………………………………………………………………………………………………..
………………………………………………………………………………………………..

HEI code	Interview platform	Interview date
RHEI1 to RHEI46	Zoom, Skype, WebEx, MS Teams	March - May 2022

Thank you!

Data collection

Data collection and analysis for the interview questionnaire

Data analysis (%)				
General Aspects				
		Yes	No	I dont know
1	Is the HEI aware of the five ESD priority areas?	84.78	4.35	10.87
2	Does the HEI consider the ESD strategies in its activities?	82.61	0.00	17.39
3	Is the HEI development based on ESD?	60.87	0.00	39.13
Advancing policy				
		Yes	No	I dont know
4	Does the HEI have a ESD framework?	47.83	10.87	41.30
5	Have you ever participated/consulted in ESD policies?	0.00	56.52	43.48
6	Do you find the policies difficult to apply?	4.35	82.61	13.04
7	Does the HEI have a systemic change plan related to ESD?	56.52	4.35	39.13
8	Does the HEI changed its policy in order to respond to emerging sustainability challenges?	52.17	8.70	43.48
9	Are ESD policies of the HEI related to the SDG 4 – Quality education?	52.17	4.35	47.83

	Transforming learning and training environments:			
		Yes	No	I dont know
10	Is the existing HEI curriculum based mainly on classic learning environment?	32.61	21.74	45.65
11	Are teachers willing to change their teaching methods for a new type of learning environment?	63.04	10.87	26.09
12	Are students willing to change their learning methods for a new type of learning environment?	97.83	2.17	0.00
13	Do the lectures inside the HEI contain sustainability principles?	26.09	23.91	50.00
14	Is the HEI considering the trends towards emerging trades in its curriculum development?	45.65	6.52	47.83
15	Are e-learnig platforms used intensively during the pandemic a key factor for ESD?	71.74	2.17	26.09
	Building capacities of educators and trainers			
		Yes	No	I dont know
16	Are the ESD values promoted among teachers?	21.74	45.65	32.61
17	Do the HEI teachers apply a sustainable principle teaching style?	28.26	2.17	69.57
18	Are the HEI teachers able to provide a digital learning environment?	63.04	2.17	34.78
19	Does the HEI need to invest in teachers' digital skills?	78.26	2.17	19.57
20	Does the HEI provide teachers the means necessary to gain knowledge about ESD?	26.09	10.87	63.04
21	Does the HEI provide the requisite motivation to address sustainable development issues?	23.91	39.13	36.96
22	Does the HEI provide ESD lectures/service trainings?	10.87	4.35	84.78
	Empowering and mobilizing youth			
		Yes	No	I dont know
23	Has the HEI implemented a youth engagement policy related to ESD?	41.30	19.57	39.13
24	Are the ESD priority areas known by students in the HEI?	63.04	10.87	26.09
25	Are students involved in HEI sustainable development campaigns?	13.04	21.74	65.22
26	Does the HEI offer SD internships?	6.52	13.04	80.43
27	Are the students in the HEI aware of the necessity of a sustainable development overall?	56.52	13.04	30.43
28	Is the HEI involving students in local community activities?	69.57	4.35	26.09
29	Is the HEI offering among students strengthening environment literacy?	50.00	15.22	34.78
30	Has the HEI presented to students the term and elements of a sustainable lifestyle?	56.52	0.00	43.48
31	Does the HEI continue certain curricula from secondary education system regarding ESD?	4.35	54.35	41.30
	Accelerating sustainable solutions at local level			
		Yes	No	I dont know
32	Does the HEI provide on a local level information regarding ESD?	4.35	45.65	50.00
33	Does the HEI provide on a local level training or campaigns for SD awareness?	2.17	50.00	47.83
34	Does the HEI R&D consider the national/regional/local social/environmental problems?	60.87	0.00	39.13
35	Has the HEI contributed to SD through its innovation?	82.61	0.00	17.39

36	Can the HEI innovations be found in the development of local level areas?	52.17	2.17	45.65
37	Is the HEI disseminating the research results among local community actors involved in SD change?	58.70	0.00	41.30
38	Has the HEI developed strategic partnerships for achieving ESD?	84.78	0.00	15.22
39	Do the students' skills currently obtained through studies correspond to contemporary societal challenges?	41.30	26.09	32.61
40	Has the HEI examples of good practice in terms of the ESD?	34.78	0.00	65.22
